# TRIM16 Promotes Osteogenic Differentiation of Human Periodontal Ligament Stem Cells by Modulating CHIP-Mediated Degradation of RUNX2

**DOI:** 10.3389/fcell.2020.625105

**Published:** 2021-01-07

**Authors:** Yi Zhao, Qiaoli Zhai, Hong Liu, Xun Xi, Shuai Chen, Dongxu Liu

**Affiliations:** ^1^Department of Orthodontics, School and Hospital of Stomatology, Cheeloo College of Medicine, Shandong University & Shandong Key Laboratory of Oral Tissue Regeneration & Shandong Engineering Laboratory for Dental Materials and Oral Tissue Regeneration, Jinan, China; ^2^Center of Translational Medicine, Zibo Central Hospital, Shandong, China

**Keywords:** TRIM16, CHIP, ubiquitination, osteogenic differentiation, hPDLSCs

## Abstract

Bone regeneration is the ultimate goal of periodontal therapies, in which osteogenic differentiation of human periodontal ligament stem cells plays a critical role. The tripartite motif (TRIM)16, an E3 ubiquitin ligase, is downregulated in periodontal tissues of patients with periodontitis, while the role of TRIM16 in the osteogenic differentiation of human periodontal ligament stem cells (hPDLSCs) is largely unknown. Firstly, we found that TRIM16 was increased throughout the osteogenic media induced differentiation of hPDLSCs. Then overexpression plasmids and specific short-hairpin RNAs (shRNAs) were constructed to manipulate the expression of target molecules. TRIM16 significantly promoted alkaline phosphatase activity, mineralized nodule formation, and positively regulated the expression of osteo-specific markers RUNX2, COL1A1 and OCN except the mRNA of RUNX2. Mechanistically, TRIM16 serves as a pivotal factor that stabilizes RUNX2 protein levels by decreasing CHIP-mediated K48-linked ubiquitination degradation of the RUNX2 protein. This study identified a novel mechanism of TRIM16 in regulating stability of the RUNX2 protein, which promoted the osteogenic differentiation of hPDLSCs. TRIM16 may be a potential target of stem cell based-bone regeneration for periodontal therapies.

## Introduction

Periodontal diseases represent a broad range of progressive inflammatory conditions characterized by irreversible attachment loss and bone resorption (Kinane et al., 2017). Bone regeneration and repair, the ultimate goal of periodontal therapies, also are known to be one of the beneficial approaches for both dental implants and orthopedic treatment (Lee et al., 2014). Stem cell-based approaches coupled with scaffolding and guiding biomaterials have been used to promote bone regeneration (Sculean et al., 2015). Mesenchymal stem cells (MSCs) isolated from the human periodontal ligament (hPDLSCs) have the ability to self-replenish and differentiate into cementoblast/osteoblasts and adipocytes *in vitro* (Seo et al., 2004). Wei et al. have shown that the periodontal tissues and bioengineered tooth root (bio-root) structure can be regenerated based on hPDLSCs in mini swine (Wei et al., 2013). This kind of tissue-specific stem cell line is therefore considered as a promising and suitable cell source for complex periodontal regeneration. In this regard, better understanding of mechanism that is involved in the osteogenic differentiation of hPDLSCs would be meaningful to develop novel therapeutic strategies to achieve periodontal tissue regeneration.

Accumulating evidence has elucidated that the ubiquitin-proteasome system plays an important role in osteogenesis (Kumar et al., 2015; Zhu et al., 2017). The ubiquitin-protein ligases (E3), the most abundant group of ubiquitination enzymes, modulates the bone formation by controlling protein levels of receptor tyrosine kinases, signaling molecules and transcription factors (Matsumoto et al., 2017; Mokuda et al., 2019). Runt-related transcription factor 2 (RUNX2) is a critical transcription factor for bone formation and osteoblast differentiation. RUNX2 protein levels has been reported to be regulated through a ubiquitin-proteasomal degradation mechanism at the post-translational level (Komori et al., 1997; Kim H.J. et al., 2020). Smurf1 interacts directly with RUNX2 and mediates RUNX2 degradation in an ubiquitin- and proteasome-dependent manner (Zhao et al., 2003; Yang et al., 2014). The carboxy terminus of HSP70 interacting protein (CHIP)/STUB1 enhanced ubiquitination and degradation of SMAD proteins, and negatively regulated RUNX2 protein stability via a ubiquitination-dependent degradation (Li et al., 2008).

TRIM16 belongs to the tripartite motif (TRIM) family of proteins, which include ∼75 proteins with E3 ligase activities and diverse functions in cell proliferation, differentiation, apoptosis, carcinogenesis and autophagy (Bell et al., 2012; Hatakeyama, 2017). TRIM16, devoid of a typical RING domain, harbors two B-box domains, a coiled-coil domain and a C-terminal domain. TRIM16 is ubiquitously expressed, and is localized predominantly in the cytosol, while it may be translocated to the nucleus in the G1 phase of the cell cycle to affect cell cycle progression and cellular differentiation in neuroblastoma (Bell et al., 2013). TRIM16 is also known as an oxidative stress-responsive protein that confers cytoprotective effects by reinforcing the intracellular antioxidant capacity during cerebral ischemia/reperfusion injury and periodontitis (Ren et al., 2020; Zhao et al., 2020). What’s more, significant downregulation of TRIM16 was observed in periodontal tissue of patients with periodontitis compared with tissues in healthy individuals (Aral et al., 2020).

Previous studies have defined the functions of TRIM16 in various biological processes; Nevertheless, its effects on osteogenesis have not been fully elucidated. TRIM16 has been shown to interact with galectin-3 by providing a platform for the assembly of core autophagic machinery in response to oxidative or proteotoxic stress (Kumar et al., 2017; Jena et al., 2019). Early study found that the expression pattern of Runt-related transcription factor 2 (Runx2) showed a positive correlation with Galectin-3 during osteoblastic development (Stock et al., 2003). Recent study has showed that TRIM16 co-regulated the osteogenic differentiation of hBMSCs with Galectin-3 (Chen et al., 2020). Thus, there may be potential association between TRIM16 and RUNX2, and the biological roles of TRIM16 in osteogenic differentiation of hPDLSCs requires further investigation.

In this study, we explored functions of TRIM16 during the osteogenic differentiation of hPDLSCs. We found that TRIM16 promoted the osteogenic differentiation of hPDLSCs by decreasing CHIP-mediated degradation of RUNX2. Therefore, targeting TRIM16 may represent a potential therapeutic strategy to promote bone regeneration in patients with periodontal disease.

## Materials and Methods

### Cell Culture

This study was approved by the Medical Ethics Committee of the School of Stomatology, Shandong University. hPDLSCs were obtained from premolars of healthy teenagers (aged 14–20) extracted for orthodontic purposes. Tissues from the middle third of the tooth root were collected, minced into small fragments of approximately 1–2 mm^3^, then digested in a solution of 3 mg/ml collagenase type I (Sigma-Aldrich, St. Louis, MO, United States) and 4 mg/ml dispase (Sigma-Aldrich) at 37°C for 1 h. The primary cells were cultured with α-MEM (Biological Industries, Beit Haemek, Israel) containing 20% fetal bovine serum (FBS) (Biological Industries) at 37°C with 5% carbon dioxide. The medium was replaced with fresh medium every 3 days until the cell monolayer reached 90% confluence. Cells were then passaged at a dilution ratio of 1:2 to expand the culture in 10% FBS medium. Cells from passages 3–7 were used for subsequent experiments.

The stemness of hPDLSCs was characterized by phenotype analysis of hPDLSCs. Cells were trypsinized and washed with PBS and then incubated with CD90-FITC, CD44-PE and PE-negative cocktail (CD45-PE, HLA-DR-PE). After incubation at 4°C protected from light for 1 h, the cells were washed with PBS and flow cytometry was performed with a BD Accuri C6 flow cytometer (BD Biosciences, San Jose, CA, United States).

The HEK293T cell line was obtained from the American Tissue Culture Collection (ATCC) and was cultured in DMEM (Sigma-Aldrich, St Louis, MO, United States) with 10% FBS and 100 μg/ml penicillin-streptoMycin sulfate (Sigma-Aldrich) in a 5% CO_2_ humidified atmosphere at 37°C.

### *In vitro* Differentiation Assays

Osteogenic induced medium contained complete culture medium supplemented with 100 nM dexamethasone (Solarbio, Beijing, China), 10 mM β-glycerophosphate (Solarbio) and 50 mg/l ascorbic acid (Solarbio). Adipogenic medium contained complete culture medium supplemented with 1 μM dexamethasone (Solarbio), 0.2 mM indomethacin (Solarbio), 0.01 g/l insulin (Solarbio) and 0.5 mM isobutyl-methylxanthine (Solarbio). Cells were cultured in 24-well plates with osteogenic differentiation medium or adipogenic differentiation medium. The medium was replaced every other day. Osteogenic differentiation was evaluated with alkaline phosphatase (ALP) and alizarin red (AR) staining (Sigma-Aldrich). Adipogenic differentiation was evaluated with Oil Red O staining (Solarbio). All methods followed protocols recommended by the manufacturers.

### Plasmid Construction and Transfection

To stably express TRIM16, the full-length open reading frame (ORF) of TRIM16 (GenBank NM_001348120) was amplified from a Human Multiple Tissue cDNA Panel (BD Biosciences) and then cloned into pLVX-puro at the *Xho*I and *Eco*RI sites. To knock down TRIM16, shRNA targeting TRIM16 and a scramble sequence were synthesized by Invitrogen (Beijing, China). The sequence of shRNA against TRIM16 and a negative control were then inserted into the pLKO.1 vector and named sh-TRIM16 and sh-NC, respectively. The transcripts of CHIP (GenBank NM_005861) and RUNX2 (GenBank NM_001024630) were amplified by PCR and subcloned into the pCMV-3Tag6 (Agilent Technologies, Santa Clara, CA, United States) and pCMV-Myc (Clontech, Mountain View, CA, United States) vectors, respectively. The constructs were named Flag-CHIP or Myc-RUNX2. All constructs were confirmed by direct sequencing (Table 1). The pRK5-HA-Ubiquitin-WT and K48, K63 were gifts from Ted Dawson (Addgene, Watertown, MA, United States; plasmid # 17608; 17605, 17606).

**TABLE 1:** Primers used in plasmid construction.

Genes	Forward (5′→3′)	Reverse (5′→3′)
TRIM16	GACTCTCGAGGCCACCATGGCGAGTTGGATCTAA	GACTGAATTCCTAGGGAGCAGTCCCCACCAAG
shTRIM16	CCGGGCAGTGAAGTCCTGTCTAACCCTCGAG GGTTAGACAGGACTTCACTGCTTTTTG	AATTCAAAAAGCAGTGAAGTCCTGTCTAACCC TCGAGGGTTAGACAGGACTTCACTGC
CHIP	AGTCGGATCCTCAGTAGTCCTCCACCCAGCCATTC	AGTCGAATTCGCCACCATGGATTACAAGGATGACGACG

### RNA Isolation and RT-qPCR Assays

Total RNA was extracted from hPDLSCs using TRIzol reagent (Thermo Fisher Scientific, Waltham, MA, United States) according to the manufacturer’s protocol. Total RNA was quantified using a NanoDrop 2000 (Thermo Fisher Scientific) and 1,000 ng of total RNA was reverse-transcribed using a PrimeScript RT reagent kit (Takara Bio, Shiga, Japan). Gene expression levels were determined by RT-qPCR using an ABI 7500 Real-Time PCR System (Thermo Fisher Scientific). RT-qPCR was performed in a 10 μl reaction volume with a TB Green PCR Core Kit (Takara Bio) according to the manufacturer’s instructions. The expression of β-actin was used for normalization. Changes in gene expression were calculated by the 2^–ΔΔ*Ct*^ method. Primer sequences are listed in Table 2.

**TABLE 2:** Primers used in RT-qPCR.

Genes	Forward (5′→3′)	Reverse (5′→3′)
RUNX2	CACTGGCGCTGCAACAAGA	CATTCCGGAGCTCAGCAGAATA
β-actin	ATGCCAACACAGTGTTGTCTGG	TACTCCTGCTTGCTGATCCACAT
SP7	ATGGCGTCCTCCCTGCTTGA	AAAGGTCACTGCCCACAGAGT
COL1A1	GGCCTAAGGGTGACAGAGGT	AGTCAGACCACGGACGCCAT

### Western Blot and Co-immunoprecipitation

Protein lysates were extracted from hPDLSCs with RIPA buffer (Solarbio) supplemented with 1% protease inhibitors (Boster Bio, Wuhan, China) and quantified by a BCA Protein Assay Kit (Thermo Fisher). Equal amounts of total protein for each sample were separated by electrophoresis at 120 V for 90 min in a 10% sodium dodecyl sulfate polyacrylamide (SDS-PAGE) gel, and then transferred to polyvinylidene fluoride (PVDF) membranes (Merck Millipore, Burlington, MA, United States) by electroblotting at 200 mA for 2 h. The membranes were blocked with 5% non-fat milk. The primary antibodies of target proteins were incubated at 4°C overnight, and secondary antibodies were incubated at room temperature for 1 h. Finally, protein expression levels were quantitated by measuring the relative intensities of the bands using ImageJ software 5.0 (National Institutes of Health, Bethesda, MA, United States).

For co-immunoprecipitation, whole-cell extracts were lysed in IP lysis buffer composed of 25 mM Tris-HCl pH 7.4, 150 mM NaCl, 1 mM EDTA, 1% NP-40 and 5% glycerol and a protease inhibitor “cocktail” (Merck Millipore). Briefly, the supernatant was collected and incubated with protein G-Agarose for 2 h to remove non-specific binding. The lysate was mixed with antibodies and gently rotated at 4°C for 4 h, then protein G-Agarose was added to the lysate for overnight incubation. The beads were washed five times with IP lysis buffer. Co-precipitated proteins were eluted with SDS-loading buffer at 95°C for 5 min and then analyzed using western blot as previous described.

### *In vitro* Ubiquitination Assay

HEK293T cells transiently transfected with the indicated plasmids were cultured for 24 h, then collected using IP lysis buffer. Antibodies were added to the lysate and gently rotated at 4°C for 4 h, then protein G-Agarose was added to the lysate at 4°C. After overnight incubation, the beads were washed five times with IP lysis buffer and the immunoprecipitated proteins were eluted with SDS-loading buffer at 95°C for 5 min, then analyzed with western blot.

### Enzyme-Linked Immunosorbent Assay (ELISA)

To further evaluate osteogenic capacity, levels of osteocalcin (OCN) were assessed by the Human Osteocalcin ELISA Kit (ab195214, Abcam, Cambridge, MA, United States) according to the manufacturer’s instructions. The optical density values were read by a microplate reader at 450 nm. Protein levels are calculated by subtracting values from the medium and standardizing them according to the number of cells.

### Immunofluorescence Staining and Co-localization Analysis

Briefly, hPDLSCs were cultured in a 12-well plate. After osteogenic induction, cells were fixed in 4% paraformaldehyde for 20 min. Then they were blocked with 5% bovine serum albumin for 30 min and incubated with anti-RUNX2 at 1:500 dilutions and anti-TRIM16 at 1:100 dilutions for overnight. Then cells were washed and incubated with fluorescence-conjugated secondary antibody Alexa Fluor594 (AB150116) and Alexa Fluor488 (ab150077) diluted 1:1,000 in blocking solution (Abcam, Shanghai, China) for 2 h at 37°C. Nuclei were stained with DAPI (40, 60-diamidino-2- phenylindole hydrochloride; Molecular Probes, Invitrogen). Cells were observed using an EVOS M5000 microscope (Thermo Fisher Scientific, American). The co-localization analysis was performed using ImageJ software 5.0 (National Institutes of Health, Bethesda, MA, United States).

### LC-MS/MS and Data Analysis

hPDLSCs were transfected with a pLVX-TRIM16 and RUNX2 lentiviral vector and collected after a 7 days incubation in osteogenic medium. After in-gel digestion, the peptides were subjected to NSI source followed by tandem mass spectrometry (MS/MS) in Q ExactiveTM Plus (Thermo) coupled online to the UPLC. A data-dependent procedure that alternated between one MS scan followed by 20 MS/MS scans with 15.0s dynamic exclusion. Automatic gain control (AGC) was set at 5E4. The resulting MS/MS data were processed using Proteome Discoverer 1.3. Peptide confidence was set at high, and peptide ion score was set > 20. The process was performed by the PTM BioLabs.

### Reagents and Antibodies

MG132, cycloheximide, and 3-MA were obtained from Sigma-Aldrich (St. Louis, MO, United States); anti-Myc, anti-Flag, anti-hemagglutinin (HA), anti-GAPDH, protein G agarose used for IP and horseradish peroxidase-conjugated secondary antibodies were from Santa Cruz Biotechnology (Dallas, TX, United States); anti-RUNX2 were obtained from Cell Signaling Technology (Beverly, MA, United States); anti-TRIM16, anti-CHIP and anti-HSP70 were from Proteintech (Rosemont, IL, United States); anti-COL1A1 was from Servicebio (Wuhan, China).

### Statistical Analysis

Each experiment was performed in triplicate. Data were presented as the means ± standard deviations. Statistical analysis was performed by Student’s *t*-test or one-way analysis of variance (ANOVA) with GraphPad software (GSL Biotech LLC, San Diego, CA, United States). *P* < 0.05 were considered statistically significant.

## Results

### Expression of TRIM16 in hPDLSCs Was Enhanced During Osteogenic Differentiation

**FIGURE 1:**
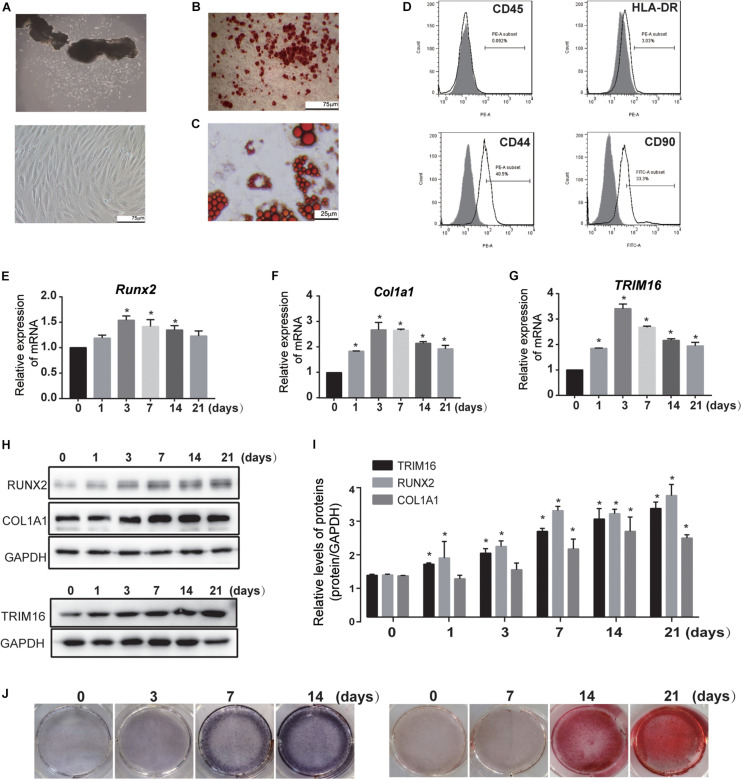
Expression of TRIM16 in hPDLSCs was enhanced during osteogenic differentiation. **(A)** hPDLSCs were derived from the periodontal ligament and cultured from passage 3 to passage 7 (*n* = 12). **(B)** Alizarin Red–positive mineralized matrix and **(C)** Oil Red O–positive lipid droplets were shown to possess multi-differentiation capacity (*n* = 3). **(D)** MSC-specific surface markers were detected by flow cytometry (*n* = 3). **(E–G)** mRNA expression of osteo-specific factors and TRIM16 was analyzed by quantitative real-time polymerase chain reaction (RT-qPCR) after induction (*n* = 3). **(H,I)** Protein expression levels of osteo-specific factors and TRIM16 were determined at indicated time after induction of osteoblastic differentiation (*n* = 3). **(J)** ALP staining and Alizarin red staining during osteogenic differentiation of hPDLSCs was examined. (*n* = 3) *P < 0.05 vs. hPDLSCs in the NC group.

hPDLSCs derived from periodontal ligament explants had a long spindle-like morphology and were arranged in whirlpool formations (Figure 1A). To determine the multi-differentiation capacity of these hPDLSCs, alizarin red–positive mineralized matrix (Figure 1B) and oil red O–positive lipid droplets (Figure 1C) were observed after osteogenic and adipogenic induction, respectively. The immunophenotype assay showed that MSC-specific surface markers (CD90, CD44) were positive, while the typical hematopoietic and endothelial cell-specific markers (CD45 and HLA-DR) were negative (Figure 1D), indicating that the primary hPDLSCs exhibited mesenchymal stem cell characteristics.

In an effort to profile TRIM16 expression patterns throughout osteogenic differentiation of hPDLSCs, we performed RT-qPCR and western blot. The results showed that TRIM16 mRNA and protein expression were enhanced during the commitment to an osteogenic linage (Figures 1E–J), suggesting that TRIM16 may be involved in the osteogenic differentiation of hPDLSCs.

### TRIM16 Promoted the Osteogenic Differentiation of hPDLSCs

**FIGURE 2:**
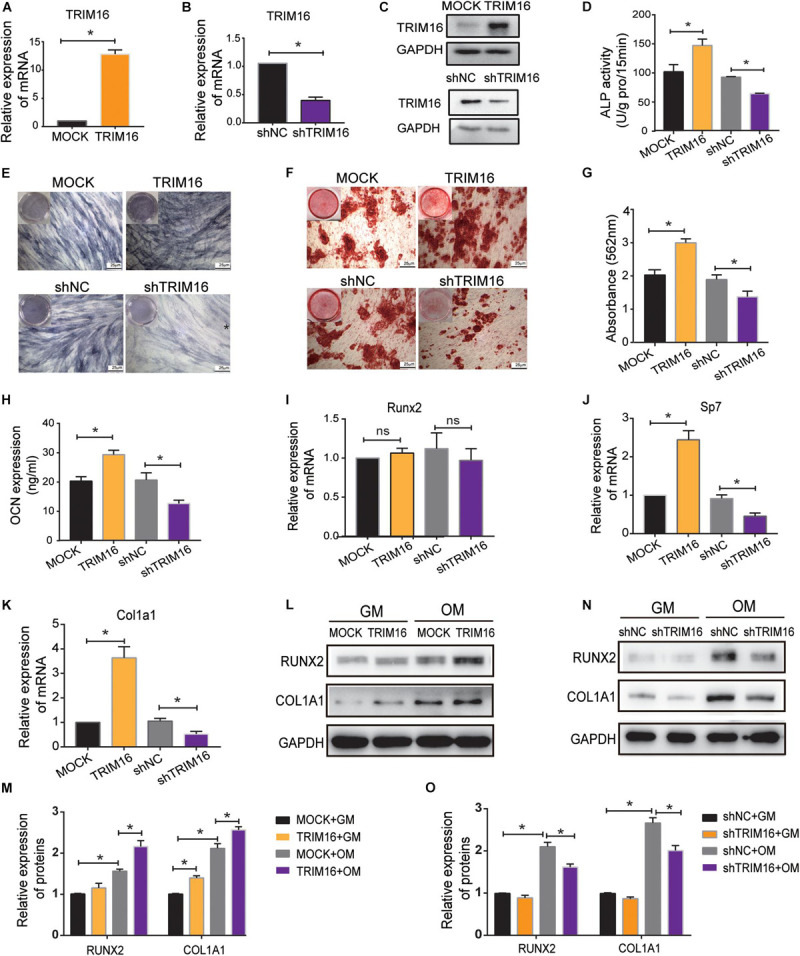
TRIM16 promoted the osteogenic differentiation of hPDLSCs. **(A,B)** The efficacy of a TRIM16-overexpressing lentiviral vector and a TRIM16 specific shRNA were measured by RT-qPCR. **(C)** The efficacy of a TRIM16-overexpressing lentiviral vector and a TRIM16 specific shRNA were measured by western blot. **(D,E)** Alkaline phosphatase activity was performed after osteogenic induction for 7 days (*n* = 3). **(F,G)** Alizarin red staining and quantity analysis was performed after osteogenic induction for 14 days. (*n* = 3) **(H)** OCN levels were measured by an ELISA assay (*n* = 3). The mRNA expression of osteo-specific markers **(I)** RUNX2, **(J)** Sp7 and **(K)** COL1A1 was assessed by RT-qPCR (*n* = 3). **(L–O)** The protein expression of RUNX2, COL1A1 was assessed by western blot after indicated treatment (*n* = 3). GM represents general medium, OM represents osteogenic-induced medium. *P < 0.05 vs. the control group, respectively.

To investigate the potential functions of TRIM16 during osteogenic differentiation of hPDLSCs, a TRIM16-overexpressing lentiviral vector was constructed and transfected into hPDLSCs. Concurrently, a TRIM16 specific shRNA was used to knockdown its expression. Efficacy was determined by RT-qPCR and western blot (Figures 2A–C). To evaluate the capacity of osteogenic differentiation, ALP staining and AR staining were performed after osteogenic induction for 7 or 14 days. The results showed that overexpression of TRIM16 significantly increased ALP activity and mineralized nodule formation, which were inhibited by knockdown of TRIM16 (Figures 2D–G). In an ELISA assay, levels of OCN were significantly higher in the presence of TRIM16 than in the control (Figure 2H). In assessment of the levels of several osteogenic-related markers, TRIM16 increased mRNA expression of SP7 and COL1A1 (Figures 2J,K) and upregulated protein expression of COL1A1 and RUNX2 (Figures 2L,M). Knockdown of TRIM16 significantly reduced mRNA expression of SP7 and COL1A1 (Figures 2J,K) and protein levels of COL1A1 and RUNX2 (Figures 2N,O). Interestingly, TRIM16 had no effects on RUNX2 mRNA expression (Figure 2I), but influenced RUNX2 protein expression significantly, indicating that TRIM16 might regulated RUNX2 at the post-transcription or post-translation levels.

### TRIM16 Reduced Ubiquitination and Degradation of RUNX2

**FIGURE 3:**
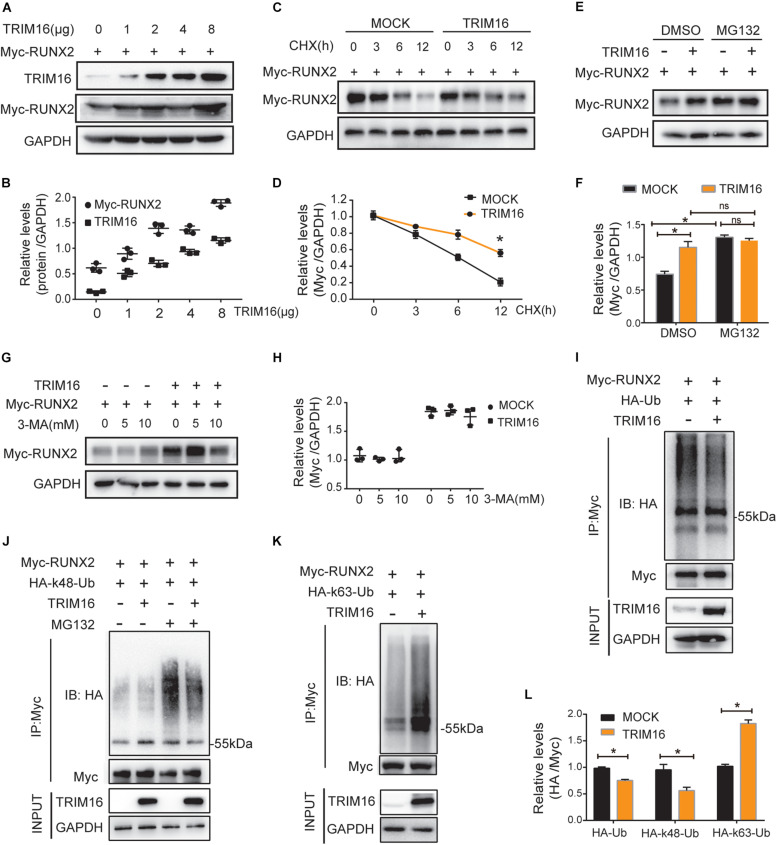
TRIM16 reduced ubiquitination and degradation of RUNX2. **(A)** Immunoblot analysis of RUNX2 in total extracts with different doses of a TRIM16 overexpression plasmid (*n* = 3). **(B)** Relative quantitative analysis of results of **(A)**, the Pearson Correlation is 0.962, *p* < 0.05. **(C,D)** Immunoblot analysis of extracts from HEK293T cells transfected with Myc-RUNX2 and a TRIM16 overexpression plasmid or control vector treated with cycloheximide (CHX) for various times. The expression of RUNX2 and TRIM16 were quantitated by measuring band intensities using ‘ImageJ’ software. Values were normalized to GADPH (*n* = 3). **(E,F)** Immunoblot analysis of extracts from HEK293T cells transfected with Myc-RUNX2 and a TRIM16 overexpression plasmid then treated with MG132 (10 mM) for 4 h (*n* = 3). **(G,H)** Immunoblot analysis of extracts from HEK293T cells transfected with Myc-RUNX2 and a TRIM16 overexpression plasmid or control vector treated with the indicated concentration of 3-MA for 4 h. Cells were transfected with Myc-RUNX 2 and a TRIM16 overexpression plasmid or control vector, HA-tagged ubiquitin (HA-Ub), HA-tagged K48-linked polyubiquitination (HA-K48-Ub), or HA-tagged K63-linked polyubiquitination (HA-K63-Ub), respectively, followed by IP with anti-Myc, and probed with anti-HA (*n* = 3). **(I)** The ubiquitination of RUNX2 influenced by TRIM16. **(J)** The K48-linked polyubiquitination of RUNX2 influenced by TRIM16. **(K)** The K63-linked polyubiquitination of RUNX2 influenced by TRIM16. **(L)** Relative quantitative analysis of the results of **(I–K)**. *P < 0.05 vs. the control group, respectively.

To verify the effects on RUNX2 expression of TRIM16, we used various doses of TRIM16 overexpression plasmid to examine the changes of RUNX2 protein levels. TRIM16 consistently increased RUNX2 protein expression in a dose-dependent manner (Figures 3A,B). The TRIM family of proteins has been implicated in the positive regulation of several critical transcription factors by reducing their ubiquitin–proteasome degradation; then, the protein biosynthesis inhibitor cycloheximide (CHX) and the proteasome inhibitor MG-132 were used for various amounts of time and concentrations to determine whether TRIM16 could inhibit protein degradation of RUNX2. To rule out whether TRIM16 inhibited RUNX2 degradation via autophagy, cells were treated with different concentrations of autophagy inhibitor 3-MA. The results showed that TRIM16 retarded the reduction of RUNX2 protein compared to the control group after treatment with CHX (Figures 3C,D), and TRIM16-induced RUNX2 stability compared with control group was attenuated by MG-132 (Figures 3E,F), which was not affected significantly by 3-MA (Figures 3G,H), indicating that TRIM16 inhibited the proteasomal degradation of RUNX2. Besides, we examined the expression of LC3B and p62 to investigate whether the autophagy was involved in TRIM16-medidated stability of RUNX2 (Supplementary Figure S1), and there was no significant difference of them between MOCK and TRIM16.

TRIM16 belongs to TRIM family proteins whose most of the members hold E3 ligase activity. So next we tested whether TRIM16 affects ubiquitination status of RUNX2. We found that overexpression of TRIM16 reduced the ubiquitination of RUNX2 (Figures 3I,L). To determine the forms of RUNX2 ubiquitination affected by TRIM16, immunoprecipitation assays were performed in the presence of two different variants, ubiquitin mutant vectors K48 (HA-K48-UB) and K63 (HA-K63-UB), which contain arginine substitutions of all of its lysine residues except the one at position 48 and 63, respectively. Overexpression of TRIM16 reduced K48-linked poly-ubiquitination of RUNX2 but increased K63-linked poly-ubiquitination of RUNX2 (Figures 3J–L).

### CHIP Was Involved in TRIM16-Mediated Osteogenic Differentiation

**FIGURE 4:**
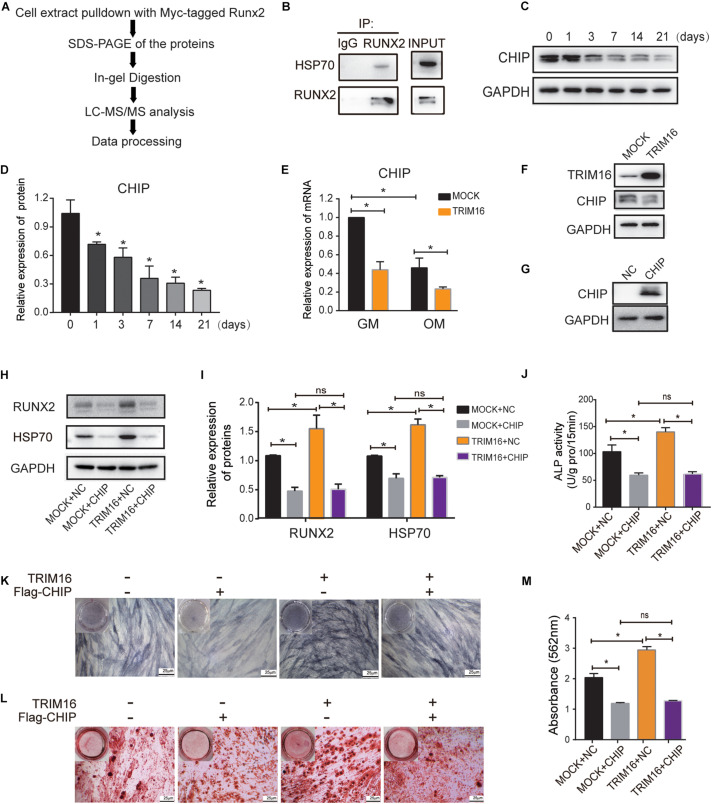
CHIP was involved in TRIM16-mediated osteogenic differentiation. **(A)** The schematic flow of LC-MS/MS analysis. **(B)** Interaction between HSP70 and RUNX2 was examined in hPDLSCs (*n* = 3). **(C,D)** Expression of CHIP during osteogenic differentiation of hPDLSCs was examined by western blot (*n* = 3). **(E)** The mRNA levels of CHIP regulated by overexpression of TRIM16 (*n* = 3). **(F)** Protein levels of CHIP regulated by overexpression of TRIM16 (*n* = 3). **(G)** The efficacy of a CHIP-overexpressing lentiviral vector was measured by western blot. **(H,I)** The expression of RUNX2 and HSP70 regulated by CHIP with or without TRIM16. **(J,K)** ALP activity and **(L,M)** Alizarin red staining was performed with indicated transfection after osteogenic induction for 7 days or 14 days (*n* = 3). *P < 0.05 vs. the control group, respectively.

Next, we sought to determine the reason for the TRIM16-mediated stability of RUNX2 through a LC-MS/MS (Figure 4A). We identified the interaction between RUNX2 and HSP70 in present of overexpression of TRIM16, the sequence from original mass spectrum was provided in Supplementary Material. Then we examined and ensured the interaction between RUNX2 and HSP70 (Figure 4B). It is noteworthy that the carboxy terminus of HSP70 interacting protein (CHIP)/STUB1 has been considered as a RUNX2-interacting protein and is a negative regulator of RUNX2 stability during commitment to the osteoblast lineage. CHIP mRNA and protein levels have been shown to decrease significantly during osteogenic differentiation in MC3T3-E1 cells. Thus, we further investigated the function of CHIP in present of TRIM16 during osteogenic differentiation of hPDLSCs. We found that the CHIP protein levels decreased significantly after osteogenic induction (Figures 4C,D). Moreover, CHIP mRNA and protein levels were reduced by TRIM16 (Figures 4E,F). Then a CHIP-overexpressing lentiviral vector transfected into hPDLSCs, the efficacy was determined by western blot (Figure 4G). As expected, CHIP suppressed the expression of RUNX2 and HSP70, and the enhanced expression of RUNX2 and HSP70 in TRIM16-overexpressing hPDLSCs was inhibited by CHIP (Figures 4H,I). In addition, overexpression of CHIP significantly attenuated ALP activity and delayed mineralized nodule formation, which were promoted by TRIM16 (Figures 4J–M). These observations indicated that CHIP was involved in TRIM16-induced promotion of osteogenic differentiation and stability of RUNX2 in hPDLSCs.

### TRIM16 Decreased CHIP-Mediated Ubiquitination Degradation of RUNX2

**FIGURE 5:**
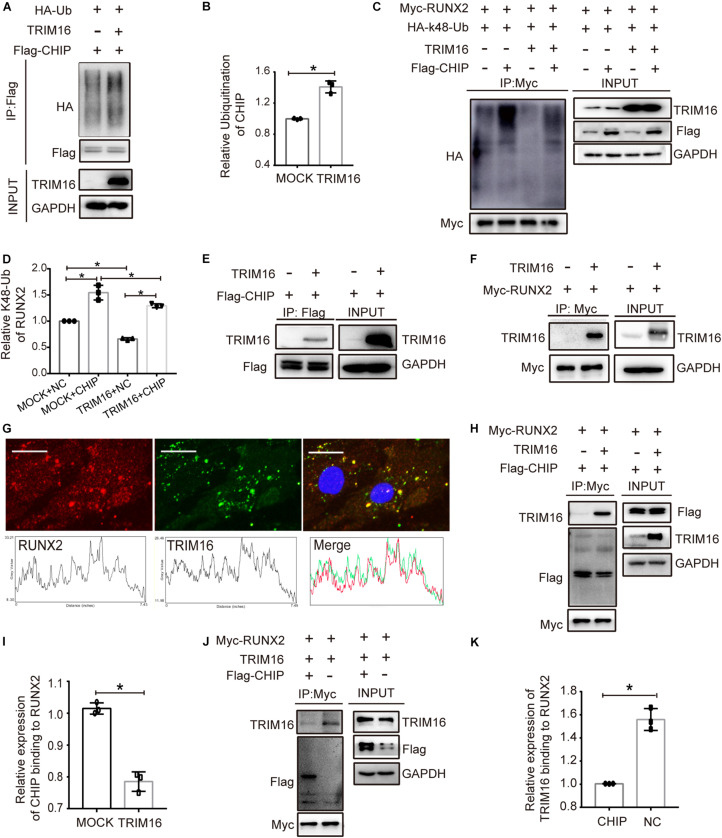
TRIM16 decreased CHIP-mediated ubiquitination degradation of RUNX2. **(A,B)** Ubiquitination of CHIP influenced by TRIM16. Immunoblot analysis of lysates from cells transfected with Flag-CHIP, HA-tagged ubiquitination (HA-Ub) with or without a TRIM16 overexpression plasmid, followed by IP with anti-Flag, and probed with anti-HA (*n* = 3). **(C,D)** Ubiquitination of RUNX2 influenced by CHIP and TRIM16. Immunoblot analysis of lysates from cells transfected with Myc-RUNX2, HA-tagged K48-linked polyubiquitination (HA-k48-Ub) with or without a TRIM16 overexpression plasmid or Flag-CHIP (*n* = 3). **(E)** Coimmunoprecipitation was performed to detect the interaction of CHIP and TRIM16. Cells were transfected with Flag-CHIP, a TRIM16 overexpression plasmid, followed by IP with anti-Flag, and probed with anti-TRIM16 (*n* = 3). **(F)** Coimmunoprecipitation was performed to detect the interaction of TRIM16 and RUNX2. Cells transfected with Myc-RUNX2, a TRIM16 overexpression plasmid, followed by IP with anti-Myc, and probed with anti-TRIM16 (*n* = 3). **(G)** Colocalization analysis between TRIM16 and RUNX2 was performed in hPDLSCs. Red fluorescence represent the expression of RUNX2, green fluorescence represents the expression of TRIM16. Scale bars, 25μm. **(H,I)** Coimmunoprecipitation was performed to detect the interaction of CHIP and RUNX2 influenced by TRIM16, followed by IP with anti-Myc, and probed with anti-Flag (*n* = 3). **(J,K)** Coimmunoprecipitation was performed to detect the interaction of TRIM16 and RUNX2 influenced by CHIP, followed by IP with anti-Myc, and probed with anti-TRIM16 (*n* = 3). *P < 0.05 vs. the control group, respectively.

**FIGURE 6:**
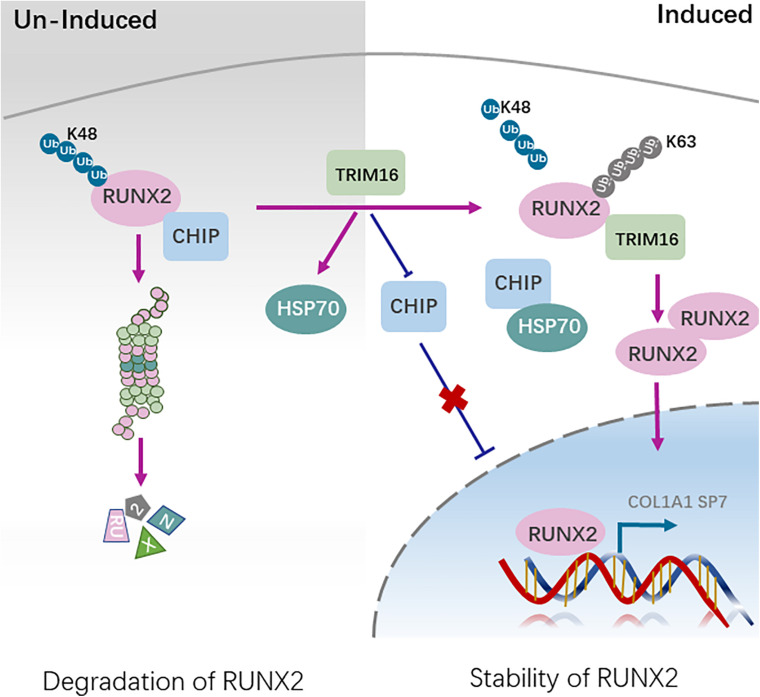
A model illustrating TRIM16 decreased CHIP-mediated ubiquitination degradation of RUNX2. During undifferentiation, CHIP interacts directly with RUNX2 and mediates RUNX2 degradation in ubiquitin- and proteasome-dependent manner. During differentiation, the enhanced expression of TRIM16 decreases CHIP-mediated RUNX2 degradation partly through interaction with RUNX2 competitively. At the same time, HSP70 expression is increased and CHIP expression is decreased by TRIM16, which further promote the osteogenic differentiation of hPDLSCs.

To study the mechanism by which CHIP implicated in stability of RUNX2 in present of TRIM16, the ubiquitination of CHIP influenced by TRIM16 and the ubiquitination of RUNX2 influenced by CHIP and TRIM16 were examined. The results showed that TRIM16 increased the ubiquitination of CHIP (Figures 5A,B), CHIP significantly increased the ubiquitination of RUNX2, and the decreased ubiquitination of RUNX2 by TRIM16 was reversed in present of CHIP (Figures 5C,D). Besides, TRIM16 physically interacts with CHIP (Figure 5E) and RUNX2 (Figures 5F,G). We also performed co-immunoprecipitation assays to detect interactions between CHIP and RUNX2 with or without overexpression of TRIM16, and the interactions between RUNX2 and TRIM16 with or without overexpression of CHIP, the results showed that the interaction between CHIP and RUNX2 was decreased by TRIM16 (Figures 5H,I), and interaction between TRIM16 and RUNX2 was also decreased by CHIP (Figures 5J,K), indicating that TRIM16 and CHIP may co-regulate RUNX2 protein expression, which is critical in the TRIM16-upregulated osteogenic differentiation capacity of hPDLSCs. Totally, TRIM16 enhanced the protein levels of RUNX2 by decreasing CHIP-mediated ubiquitination degradation of RUNX2. To illustrate the mechanism that TRIM16-mediated promotion of osteogenic differentiation in hPDLSCs, a model diagram is shown in Figure 6.

## Discussion

Previous studies have documented the functions of TRIM16 mainly in cancer, innate immune and other physiological and pathophysiological processes (Hatakeyama, 2017; Sutton et al., 2019). To date, the role of TRIM16 in osteogenesis is largely unknown. In this study, we reported a novel role for TRIM16 in osteogenic differentiation of hPDLSCs. Throughout the osteogenic media induced differentiation of hPDLSCs, the expression of TRIM16 was upregulated gradually and overexpression of TRIM16 promoted the osteogenic differentiation which was impaired by knockdown of TRIM16. All these results indicate that TRIM16 may act as a positive regulator in the osteogenic differentiation of hPDLSCs.

RUNX2 is a key transcription factor in osteogenesis and is essential for osteogenic differentiation of suture mesenchymal cells and osteoblast progenitors. Germline deletion of RUNX2 (RUNX2−/−) or inhibition of its function in mice often results in reduced numbers of osteoblasts and marked inhibition of mineralized bone formation and chondrocyte maturation (Otto et al., 1997; Maruyama et al., 2007). Therefore, stabilization of RUNX2 is an important intracellular event for both intramembrane and endochondral bone formation (Kim J.M. et al., 2020). The activity and stability of RUNX2 are modulated at the post-transcription and post-translation levels by modifications such as phosphorylation, acetylation (Gomathi et al., 2020; Kim H.J. et al., 2020; Komori, 2020). In this study, we showed that RUNX2 protein expression was significantly enhanced in the presence of TRIM16 even though RUNX2 mRNA expression remained unchanged, suggesting that posttranscriptional or posttranslational modification may be involved in TRIM16-mediated regulation of RUNX2. Recent studies have showed that TRIM16 functioned prominently as a regulator–receptor for the assembly of core autophagic machinery to modulate the selective autophagy (Kimura et al., 2017). Nevertheless, TRIM16-mediated effects on RUNX2 protein expression were not altered significantly by the autophagy inhibitor 3-MA, ruling out the possibility that TRIM16 regulates RUNX2 via an autophagy-dependent pathway. It is noteworthy that TRIM16 retards the reduction of RUNX2 protein relatively compared with the control group after treatment with the protein biosynthesis inhibitor CHX, and TRIM16-induced RUNX2 stability compared with control group was attenuated by the proteasome inhibitor MG-132. Hence, highly dynamic post-translational modification may participate in TRIM16-associated regulation of RUNX2 by the ubiquitin-proteasome system, which plays an important role in controlling the stability and activity of many proteins (Zusev and Benayahu, 2008; Song et al., 2020).

TRIM16 belongs to the TRIM family of proteins with E3 ligase activity. Several E3 ligase have been reported to play important roles in bone formation by regulating the osteogenic-related proteins. For example, Smurf1 was initially identified as a negative regulator of Smads homolog through ubiquitination and degradation of Smads, which also negatively regulate the BMP signaling pathway in osteoblastic differentiation and bone formation (Yamashita et al., 2005). Another research also demonstrated that inhibition of osteoblastic Smurf1 could be a precision medicine-based bone anabolic strategy for specific subgroup of age-related osteoporotic individuals (Liang et al., 2018). The E3 ubiquitin ligase NEDD4 is considered crucial for osteoblast maturation and necessary for intramembranous bone formation by degrading the aberrant opposite pSmad1 activated by TGFβ1, potentiating pSmad2 and increasing the gene expression of Tgfb1 by pERK1/2 (Jeon et al., 2018). TRAF4 is abnormally decreased in osteoporosis patients and modulates the osteogenic process of MSCs by acting as an E3 ligase to mediate the ubiquitination of smurf2 and cause degradation (Li et al., 2019). WWP2 facilitated the transactivation of RUNX2 by affecting the mono-ubiquitination during osteogenic differentiation (Zhu et al., 2017). Unparalleled, in our study, the forced expression of RUNX2 depends on the change of ubiquitination. We found that TRIM16 decreased the ubiquitination of RUNX2 significantly than the control group. Usually, protein degradation is the most recognized function of ubiquitination, while the effects of ubiquitination may vary considerably depending on the topological structure of the ubiquitin chains. Seven lysine residues (K6, K11, K27, K29, K33, K48, and K63) and the N-terminal methionine residue are capable of acting as receptors for conjugation, producing polyubiquitin chains, which produce different ubiquitination results in different substrates (Swatek and Komander, 2016). K48-linked and k63-linked poly-ubiquitination of proteins are two principal delivery signals in the ubiquitination system. The K48-linked poly-ubiquitination of a protein is known to serve as signals for proteasomal degradation and the K63-linked poly-ubiquitination mediate various functions such as protein re-localization, proteins oligomerization, protein stability, and activation of signaling pathways (Erpapazoglou et al., 2014; Choi et al., 2016; van Huizen and Kikkert, 2019). TRIM16 decreased K48-linked poly-ubiquitination of RUNX2, which is a known principal delivery signal for proteasomal degradation. In previous studies, TRIM16 has been reported to decrease K48-linked poly-ubiquitination and increase K63-linked poly-ubiquitination of NRF2. TRIM16 facilitated displacement and degradation of KEAP1, which is a negative regulator of NRF2 stability (Jena et al., 2018). Then we inferred that there was a negative regulator of RUNX2 modulated by TRIM16.

Several ubiquitin E3 ligases, including SMURF1, CHIP and WWP1 have been reported to induce ubiquitin-dependent degradation of RUNX2 during commitment to the osteoblast lineage (Li et al., 2008; Shu et al., 2013; Shimazu et al., 2016). In our study, we identified the interaction between RUNX2 and HSP70 in present of overexpression of TRIM16. In previous studies, HSP70 also plays an important role in cell differentiation and tissue development, including in osteogenic differentiation (Loones and Morange, 1998; Lui and Kong, 2007; Sayed et al., 2019). HSP70 knockdown impairs osteogenic and chondrogenic differentiation in human mesenchymal stem cells (Li et al., 2018). The carboxy terminus of HSP70 interacting protein (CHIP)/STUB1, a cochaperone protein interacted with Hsc/Hsp70, is identified a RUNX2-interacting protein and a negative regulator of RUNX2 stability with E3 ligase (Ballinger et al., 1999; Jiang et al., 2001; Li et al., 2008). CHIP protein levels decrease steadily accompanied by an increase in RUNX2 protein levels and restrict osteogenic differentiation. CHIP maintains the low levels of Runx2 protein in preosteoblasts and is turned down when differentiation starts to ensure that Runx2 protein can be quickly elevated to the level high required to initiate further osteoblast differentiation (Li et al., 2008). CHIP also facilitates the degradation of Smad proteins, which are critical mediators in activation of BMP signaling (Xin et al., 2005). On one hand, TRIM16 decreased the expression of CHIP and increased the ubiquitination of CHIP. Besides, overexpression of CHIP attenuated the osteogenic capacity of TRIM16-overexpressing hPDLSCs by diminishing the levels of RUNX2 and increasing its K48-linked poly-ubiquitination, which indicated that the TRIM16-mediated stability of RUNX2 is attributable to the suppression of CHIP-mediated RUNX2 proteasomal degradation. At the same time, we found that there was an increase in HSP70 by TRIM16, which confers beneficial roles in osteogenic differentiation. CHIP has been proposed to ubiquitinate the chaperones’ client proteins and targets them to the proteasome for degradation (Connell et al., 2001), and the negative relation between CHIP and HSP70 has also been observed (Zhao et al., 2012), which is consist with our results. On the other hand, both TRIM16 and CHIP interact with RUNX2. The binding between CHIP and RUNX2 was prevented by TRIM16, which is essential for K48-linked poly-ubiquitination and subsequent degradation, providing an explanation on the stability of RUNX2 by TRIM16. Another reason for the decreased interaction between them may be also related to the increased HSP70. Of note, the binding between TRIM16 and RUNX2 was also inhibited by CHIP, which could be a critical component of their interrelationship. TRIM16 might cooperate with CHIP to regulate the expression of RUNX2 during the osteogenic differentiation. The concrete underlying mechanism may cover a complex network regulation. Our research provides a new sights on the roles of TRIM16 during the osteogenic differentiation of hPDLSCs.

## Conclusion

Collectively, TRIM16 promoted osteogenic differentiation of hPDLSCs by modulating CHIP-mediated ubiquitination and protein degradation of RUNX2. Therapeutic targeting of TRIM16 may be a promising strategy for bone regeneration in periodontal therapy.

## Data Availability Statement

The original contributions presented in the study are included in the article/Supplementary Material, further inquiries can be directed to the corresponding author/s.

## Ethics Statement

The studies involving human participants were reviewed and approved by the Medical Ethics Committee of the School of Stomatology, Shandong University. Written informed consent to participate in this study was provided by the participants’ legal guardian/next of kin.

## Author Contributions

DL conceived the project design and conception. YZ and QZ were responsible for the experimental design and application including data acquisition and analysis. YZ and HL wrote and revised the manuscript. XX and SC standardized the figures. All authors gave final approval of the manuscript and agreed to be accountable for all aspects of the work.

## Conflict of Interest

The authors declare that the research was conducted in the absence of any commercial or financial relationships that could be construed as a potential conflict of interest.
