# Supplementary figures and images for: TRIM16 Promotes Osteogenic Differentiation of Human Periodontal Ligament Stem Cells by Modulating CHIP-Mediated Degradation of RUNX2
Source: Front Cell Dev Biol. 2021 Jan 7;8:625105. doi: 10.3389/fcell.2020.625105 (PMC7817816; doi:10.3389/fcell.2020.625105)

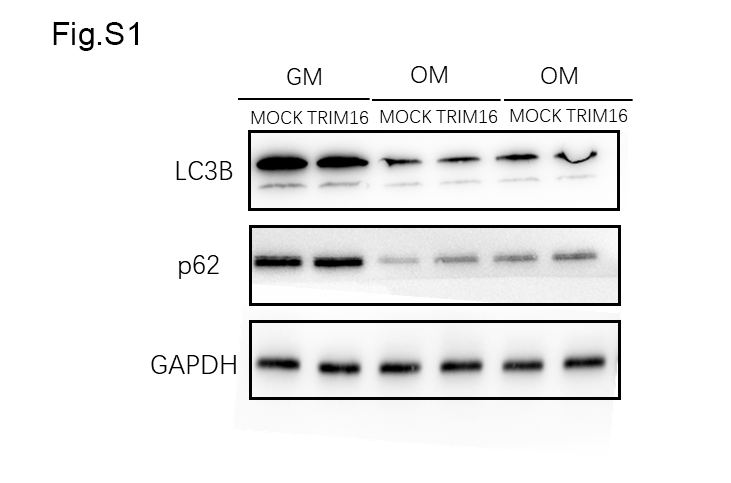

Supplement: Supplementary file 1 [file Image_1.TIF]
