# Supplementary material for: TRIM16 Promotes Osteogenic Differentiation of Human Periodontal Ligament Stem Cells by Modulating CHIP-Mediated Degradation of RUNX2
Source: Front Cell Dev Biol. 2021 Jan 7;8:625105. doi: 10.3389/fcell.2020.625105 (PMC7817816; doi:10.3389/fcell.2020.625105)
Supplement: Supplementary file 2 [file Table_1.docx]

| Accession | | Protein names | Sequence coverage(%) | Protein score | Unique peptides |
| --- | --- | --- | --- | --- | --- |
| Q13950  P11142  P11021  P0DMV9  Q14192  Q86YZ3  P68104  P21980 | Runt-related transcription factor 2  Heat shock cognate 71 kDa protein  Endoplasmic reticulum chaperone BiP  Heat shock 70 kDa protein 1B  Four and a half LIM domains protein 2  Hornerin  Elongation factor 1-alpha 1  Protein-glutamine gamma-glutamyltransferase 2 | | \| 33.78 \| \| --- \|   27.86  32.26  25.74  34.05  9.93  17.32  9.46 | 874.11  842.19  678.10  485.18  365.32  326.26  307.69  269.26 | 15  13  16  12  8  8  6  5 |

Table S1 Identification of proteins that have potential interaction with Runx2
