# Supplementary material for: TRIM16 Promotes Osteogenic Differentiation of Human Periodontal Ligament Stem Cells by Modulating CHIP-Mediated Degradation of RUNX2
Source: Front Cell Dev Biol. 2021 Jan 7;8:625105. doi: 10.3389/fcell.2020.625105 (PMC7817816; doi:10.3389/fcell.2020.625105)
Supplement: Supplementary file 3 [file Table_2.DOCX]

Sequence: NQVALNPQNTVFDAK, Charge: +2, Monoisotopic m/z: 829.92816 Da (-0.34 mmu/-0.41 ppm), MH+: 1658.84905 Da, RT: 13.54 min

Sequence: DAGVIAGLNVLR, Charge: +2, Monoisotopic m/z: 599.35156 Da (+0.39 mmu/+0.65 ppm), MH+: 1197.69585 Da, RT: 17.70 min

Sequence: TTPSYVAFTDTER, Charge: +2, Monoisotopic m/z: 744.35480 Da (+0.5 mmu/+0.68 ppm), MH+: 1487.70232 Da, RT: 12.73 min

Sequence: LLQDFFNGR, Charge: +2, Monoisotopic m/z: 555.29071 Da (+0.14 mmu/+0.25 ppm), MH+: 1109.57414 Da, RT: 16.16 min

Sequence: AFYPEEISSMVLTK, Charge: +2, Monoisotopic m/z: 807.90680 Da (-1.06 mmu/-1.31 ppm), MH+: 1614.80632 Da, RT: 18.96 min

Sequence: VEIIANDQGNR, Charge: +2, Monoisotopic m/z: 614.81793 Da (+0.24 mmu/+0.4 ppm), MH+: 1228.62859 Da, RT: 8.71 min

Sequence: AQIHDLVLVGGSTR, Charge: +2, Monoisotopic m/z: 733.41010 Da (+0.35 mmu/+0.47 ppm), MH+: 1465.81291 Da, RT: 12.39 min

Sequence: YKAEDEVQR, Charge: +2, Monoisotopic m/z: 569.28070 Da (+0.3 mmu/+0.53 ppm), MH+: 1137.55412 Da, RT: 5.47 min

Sequence: ATAGDTHLGGEDFDNR, Charge: +2, Monoisotopic m/z: 838.36847 Da (-0.55 mmu/-0.65 ppm), MH+: 1675.72966 Da, RT: 8.53 min

Sequence: DNNLLGR, Charge: +2, Monoisotopic m/z: 401.21408 Da (-0.25 mmu/-0.62 ppm), MH+: 801.42088 Da, RT: 9.06 min

Sequence: HWPFQVINDGDKPK, Charge: +3, Monoisotopic m/z: 560.95502 Da (+0.39 mmu/+0.69 ppm), MH+: 1680.85050 Da, RT: 12.85 min

Sequence: SAVEDEGLK, Charge: +2, Monoisotopic m/z: 474.23715 Da (-0.52 mmu/-1.1 ppm), MH+: 947.46703 Da, RT: 7.19 min

Sequence: IINEPTAAAIAYGLDR, Charge: +3, Monoisotopic m/z: 563.30524 Da (-0.09 mmu/-0.17 ppm), MH+: 1687.90116 Da, RT: 16.78 min

Sequence: VQVSYK, Charge: +2, Monoisotopic m/z: 362.20404 Da (-1.39 mmu/-3.84 ppm), MH+: 723.40080 Da, RT: 6.84 min
